# Quantum Spin Detection in Microfiltration Immunoassays for Ultrasensitive and High-Throughput Diagnostics

**DOI:** 10.1021/acs.analchem.5c05569

**Published:** 2026-02-06

**Authors:** Trong-Nghia Le, Xuan Mai Lam, Yi-Xiu Tang, Yuen Yung Hui, An-Jie Liu, Huan-Cheng Chang

**Affiliations:** † Institute of Atomic and Molecular Sciences, 38017 Academia Sinica, Taipei 106, Taiwan; ‡ Department of Chemical Engineering, National Taiwan University of Science and Technology, Taipei 106, Taiwan; § Department of Chemistry, 427108 National Taiwan Normal University, Taipei 106, Taiwan

## Abstract

This study demonstrates the transformative potential of quantum technologies for healthcare diagnostics by developing a new analytical method, the quantum-enabled microfiltration immunoassay (QEMFIA). QEMFIA integrates the strengths of dot blot and enzyme-linked immunosorbent assays, enabling rapid, sensitive, and quantitative detection of clinically relevant antigens using nanoscale quantum sensors in a high-throughput format. The assay leverages fluorescent nanodiamonds (FNDs) with nitrogen-vacancy centers as reporters, combined with magnetically modulated fluorescence (MMF) for background-free detection of optically addressable spin defects. Additionally, to achieve high-throughput operation, the assays are performed on a 24-well microfiltration manifold, with target antigens captured by antibodies immobilized on a nitrocellulose membrane, followed by detection using antibody-conjugated FNDs. Finally, retained FNDs are directly analyzed on the membrane via MMF under a fluorescence microscope. The limits of detection for disease markers, such as C-reactive protein and interleukin-6, are below 100 fM within 1 h. The method is compatible with standard 96-well plates and conventional lab workflows. It also supports integration with automation platforms for high-throughput analysis across a broad range of target antigens using the FND quantum sensors.

## Graphical abstract

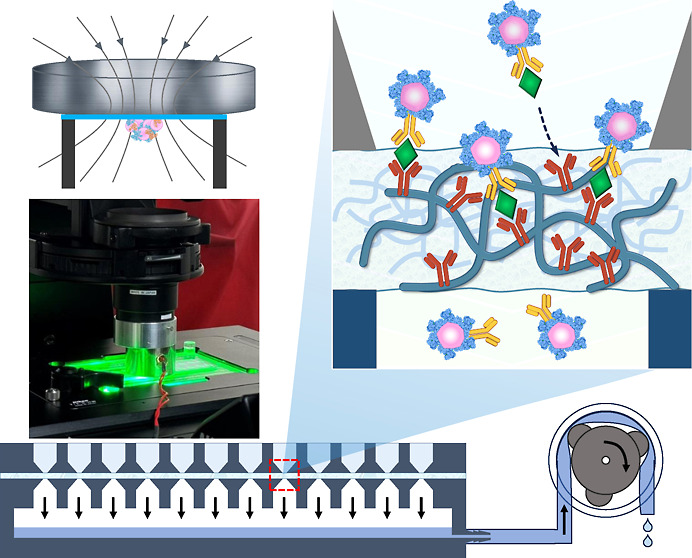

## Introduction

Quantum sensing represents a rapidly advancing frontier in measurement science, leveraging materials with unique quantum properties to achieve exceptional precision and sensitivity. Among the most promising quantum sensors are nitrogen-vacancy (NV) centers in diamonds, which have found wide-ranging applications across science and engineering.^1–6^ Fluorescent nanodiamonds (FNDs) are diamond nanoparticles notable for their high density of vacancy-related defects.^2^ These atomic-scale point defects can emit strong fluorescence in multiple colors when excited by visible light. For FNDs measuring 100 nm or larger, their fluorescence characteristics are largely unaffected by external factors, such as surface modifications or pH fluctuations in solution, because the defects are mainly deeply embedded within the chemically inert diamond lattice. Moreover, the carbon-based nanomaterials are nontoxic and their fluorescence is exceptionally photostable, making FNDs a valuable tool for reliable, high-precision quantitative analysis in biomedicine.

The negatively charged nitrogen-vacancy (NV^–^) centers in diamonds have recently garnered tremendous attention for quantum sensing applications owing to their outstanding magneto-optical properties. Specifically, the center consists of electronic spin states that can be optically addressed. Selective detection of the center’s fluorescence can be achieved by combining microwave or magnetic modulation with green light excitation. One emerging application of these quantum sensors is the use of antibody-conjugated FNDs with NV centers as immunosensors in healthcare.^7–13^ Miller et al.^7^ pioneered the application of FNDs as fluorescent reporters in lateral flow immunoassays (LFIA), enabling highly sensitive diagnostics for human immunodeficiency virus through microwave-assisted detection. Independently, Hui et al.^8^ developed a magnetically modulated fluorescence (MMF) technique for background-free detection of these bioconjugated FNDs in highly dilute solutions and also on LFIA membrane strips. In these two studies, a background rejection ratio greater than 1000 was readily established by lock-in amplification. Utilizing the spin-enhanced LFIA platform, Hsiao et al.^9^ demonstrated that this assay could effectively and quantitatively detect the nucleocapsid and spike proteins of wild-type SARS-CoV-2 and its variants during the COVID-19 pandemic. Taking an entirely different approach, Li et al.^14^ monitored variations in the spin relaxation times of NV^–^ centers in FNDs as SARS-CoV-2 RNA bound to the nanoparticles surface-conjugated with c-DNA-Gd^3+^ complexes, achieving semiquantitative analysis.

While large-scale LFIA testing for public health has proven its feasibility throughout the COVID-19 pandemic,^15^ implementing this method with high throughput for practical clinical applications remains a significant challenge.^16–18^ To address this issue, Huynh et al.^17^ developed an integrated system comprising a liquid-handling robot, an image-acquisition camera, and a multicassette holder for high-throughput LFIA testing. The robot could manage up to 96 strips in cassettes simultaneously, but the setup was bulky and had not yet gained widespread adoption. A more effective approach for high-throughput immunodiagnostics is to utilize a detection system in an 8 × 12 or 96-well format that can be operated manually and is compatible with existing automation technologies.^19–21^ However, to the best of our knowledge, no quantum sensing methods have been effectively integrated into these systems to facilitate their practical, real-world applications.

Here, we present the development of a new platform, called quantum-enabled microfiltration immunoassay (QEMFIA), for the rapid and sensitive detection of antigens or antibodies in solutions using FNDs as optical reporters. The platform is a multiwell format of vertical-flow or flow-through immunoassays,^20–25^ with enhanced sensitivity achieved through the incorporation of quantum-sensing technologies. We evaluated the performance of QEMFIA using inflammatory biomarkers, such as C-reactive protein (CRP) and interleukin 6 (IL-6), and found that the quantum-enabled platform offered enhanced sensitivity, ease of use, and higher throughput than conventional methods. Specifically, QEMFIA presents several advantages over the enzyme-linked immunosorbent assay (ELISA), the gold standard method in the field, including increased speed, fewer operational steps, and reduced reagent consumption. Furthermore, QEMFIA outperforms the dot blot assay, another widely used molecular biology technique,^26^ in terms of detection speed, quantification accuracy, and compatibility with automated workflows.

## Experimental Section

### Production of FNDs

FNDs were created by ion or electron irradiation of synthetic type-Ib diamond powders (Micron+ MDA, Element Six), followed by vacuum annealing at 800 °C and air oxidation at 450 °C, as previously described.^11^ To enhance their suitability as in vitro diagnostic reagents, the FNDs were further treated in molten KNO_3_ salts to produce particles with more uniform shapes and sizes. Briefly, 100 mg of FNDs were thoroughly mixed with 2 g of KNO_3_ and heated at 500 °C for 1 h. The mixture was then cooled to room temperature and washed with distilled deionized water, after which the rounded FND particles were surface-functionalized with carboxyl groups via acid washes in a 3:1 (v/v) H_2_SO_4_–HNO_3_ mixture at 100 °C. The FNDs prepared in this manner were negatively charged, with hydrodynamic diameters of around 100 nm as determined by dynamic light scattering.^11^

### Preparation of b-BSA- and Antibody-Conjugated FNDs

Antibody-conjugated FNDs were prepared by mixing FNDs (1 mg/mL) and antibodies (0.2 mg/mL) at a weight ratio of 5:1 and incubating at 37 °C for 30 min.^11^ Afterward, a solution containing 3% bovine serum albumin (BSA) in deionized water was added to block the nonbinding sites on the FND surface, followed by incubation for another 30 min. The mixture was then centrifuged at 20,000*g* for 10 min, and the pellets were washed twice with phosphate-buffered saline (PBS) to remove unbound antibodies. Finally, the obtained pellets (i.e., antibody-conjugated FNDs) were dispersed in 3% BSA/PBS with a final concentration of 100 μg/mL. The antibody-conjugated FND suspensions were stored at 4 °C and filtered through a 1.2 μm PET syringe filter to remove aggregates before use.

A similar procedure was employed to obtain b-BSA-conjugated FNDs, with biotinylated BSA (b-BSA) replacing the antibodies during the initial mixing step. Dynamic light scattering, which measured the hydrodynamic diameters and zeta potentials of these nanoparticle bioconjugates, confirmed successful sample preparation, as previously reported in refs 8 and 11 .

### QEMFIA Platform

Figure 1a–c illustrate the workflow for sandwich QEMFIA and provide a schematic diagram of the experimental setup. The platform consists of (1) a homemade dot-blot microfiltration manifold, featuring 24 wells on both the top sample and bottom filtration plates, (2) an unbacked lateral flow nitrocellulose (NC) membrane with a specified pore size of 12 μm and a thickness of 105–140 μm (10400012, AE100, Whatman), (3) a gasket made of Parafilm (AM-PM996, Amcor) precisely cut using a CO_2_ laser (Beamo, FLUX), and (4) a digital peristaltic pump (DP.01, Lefo Science) with a flow range of 0.06–36 mL/min (1–600 rpm). Figure S1 provides an exploded view of the microfiltration manifold and its connection to a peristaltic pump.

**Figure 1:**
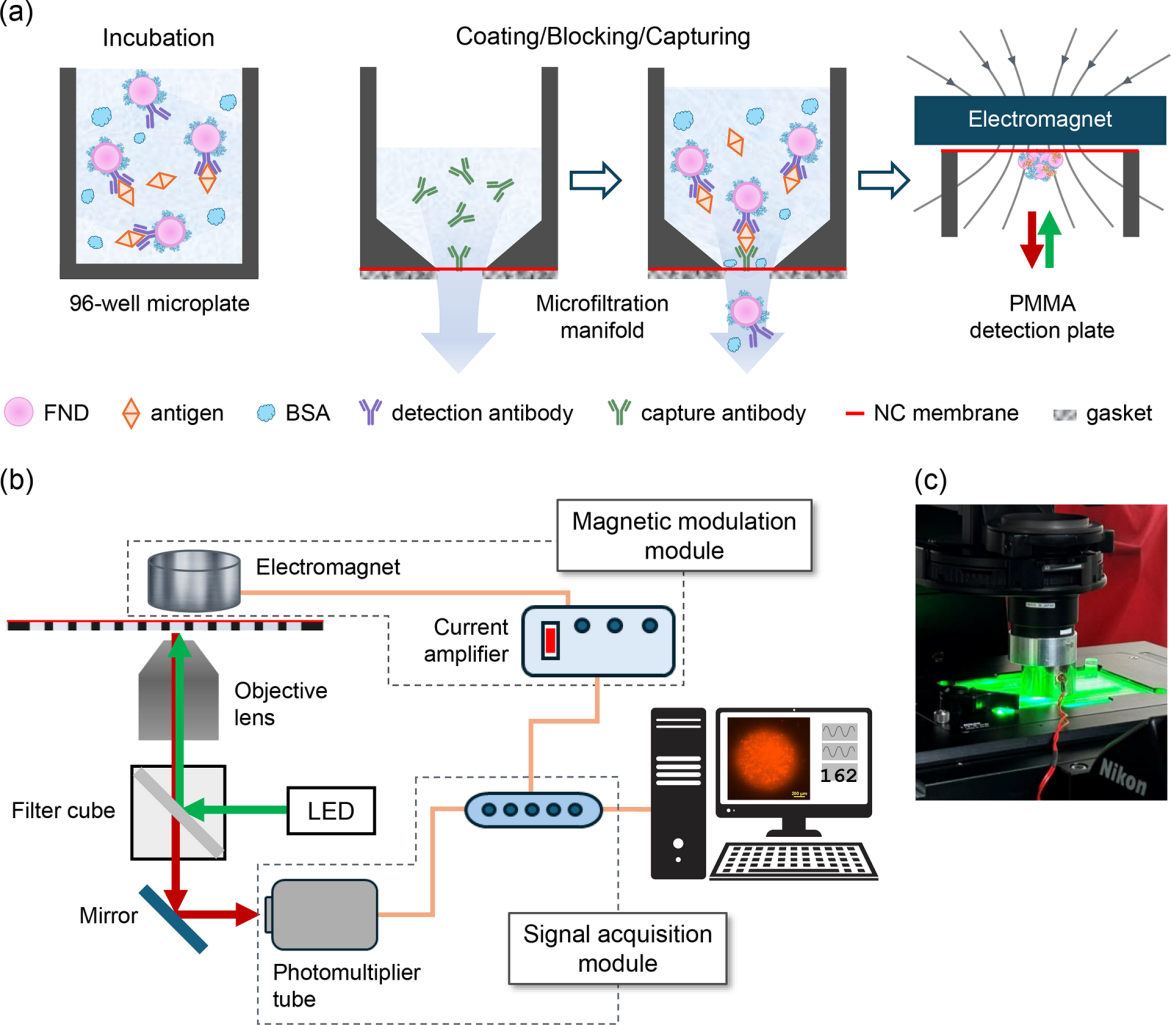
(a) Materials and methods used in sandwich QEMFIA. (b) Integration of magnetic modulation and signal acquisition modules with a standard inverted fluorescence microscope system. Details of the protocols and the setup are provided in the Experimental Section. (c) Photograph of a 24-well detection plate with an electromagnet positioned above it. The electromagnet can be easily moved in and out of the sample position by tilting the diascopic illuminator at the top by approximately 30°. The clear acrylic plate is situated on the motorized sample stage of an inverted microscope for microplate imaging and automated fluorescence intensity measurements (Video S1).

In addition to the microfiltration manifold, a 24-well detection plate, with a thickness of 3 mm and a 6 mm-diameter through-hole in each well, was fabricated for MMF measurements. Both the vacuum manifold and the 24-well plate were precision-machined from clear acrylic (poly­(methyl methacrylate) (PMMA)). The distances between wells on all the plates were 9 mm, closely matching those of a standard 96-well microplate. The transfer of the NC membrane from the dot-blot apparatus to the detection plate can be easily accomplished using double-sided tape. The design significantly simplifies integration with existing automation technologies, making it easy for high-throughput immunodiagnostic applications.

The detection system was constructed by integrating an inverted fluorescence microscope (Eclipse Ti2-E, Nikon), a magnetic modulation module, and a signal acquisition module (USB-4431, National Instruments), as illustrated in Figure 1b. During the measurements, the FND-captured NC membrane was exposed to green LED light (534 ± 30 nm) focused on the hole of each well with a 10× objective lens (Nikon). The beam diameter at the focus was about 4 mm, sufficient to excite all the particles confined within the holes. The FNDs emitted red fluorescence upon the green light excitation. The objective lens collected the fluorescence emission, which then passed through a microscope filter cube and was detected by a photomultiplier tube (PMT1001, Thorlabs). Magnetic modulation was applied to the samples using a round permanent electromagnet (EML35 mm-24, APW) positioned above the NC membrane (Figure 1). The electromagnet, with a 35 mm diameter, was driven by a homemade current amplifier at a resonant frequency of 102.4 Hz to achieve the highest possible signal levels. The signals were electronically processed, converted to digital outputs, and analyzed using a standard lock-in method with a home-built LabVIEW program.^8^ The typical data acquisition time for each MMF measurement was 30 s.

### Dispersed Fluorescence Spectra

Photoluminescence spectra of FNDs deposited on the NC membrane were acquired using a photonic multichannel analyzer (C7473, Hamamatsu) attached to the eyepieces of the inverted fluorescence microscope housing the samples. Fluorescence signals were recorded using the multichannel analyzer in the presence or absence of external magnetic fields.

### Direct QEMFIA with the Biotin–Avidin Model

Protocols include: (1) Assemble the microfiltration manifold and connect it to a peristaltic pump, as shown in Figure S1. (2) Add NeutrAvidin (NA) solution (1.5 μL, 1 mg/mL) into the individual wells of the microfiltration manifold and allow it to air-dry for 5 min. (3) Add 3% BSA/PBS (100 μL) in the individual wells, and switch on the peristaltic pump to facilitate the flow of the solution at a rate of about 20 μL/min through the membrane to fully block unbound sites on the NC membrane. (4) Switch off the pump and add b-BSA-FND suspensions (100 μL) with varying concentrations into the BSA-blocked wells. (5) Switch on the peristaltic pump to pull the reagents across the NC membrane at a rate of about 10 μL/min. (6) Wash the membrane with 1% BSA/PBS (200 μL) at a flow rate of about 20 μL/min. (7) Transfer the NC membrane with b-BSA-FNDs captured by the immobilized NA to the homemade plate using double-sided tape. (8) Measure MMF of FNDs on an inverted fluorescence microscope equipped with an electromagnet.

### Sandwich QEMFIA of CRP

Polyclonal anti-CRP mouse antibody pAb-P17 served as both capture and detection antibodies. Protocols include: (1) Add pAb-P17-FND suspension (2 μg/mL, 100 μL) to CRP solution (100 μL) with varying concentrations in the individual wells of a standard 96-well microplate, and incubate for 15 min. (2) Assemble the microfiltration manifold and connect it to a peristaltic pump, as shown in Figure S1. (3) Add pAb-P17 solution (1.5 μL, 1 mg/mL) into the individual wells of the microfiltration manifold and allow it to air-dry for 5 min. (4) Add 3% BSA/PBS (100 μL) in the individual wells, and switch on the peristaltic pump to facilitate the flow of the solution at a rate of about 20 μL/min through the membrane to fully block unbound sites on the NC membrane. (5) Transfer the CRP-pAb-P17-FND solution prepared in Step 1 to the microfiltration manifold, and switch on the peristaltic pump to pull the reagents across the NC membrane at a rate of about 10 μL/min. (6) Wash the membrane with 1% BSA/PBS (200 μL) at a flow rate of about 20 μL/min. (7) Transfer the NC membrane with CRP-pAb-P17-FNDs captured by the immobilized pAb-P17 to the homemade plate using double-sided tape. (8) Measure MMF of FNDs on an inverted fluorescence microscope equipped with an electromagnet.

### Sandwich QEMFIA of CRP in Human Serum

Human serum was filtered using a 1 μm membrane and subsequently diluted in 3% BSA/PBS with dilution factors ranging from 1:1000 to 1:1,000,000. A 100 μL volume of the diluted serum was used for the assay, following the same procedures described above. For spike-in experiments, CRP was added to the filtered human serum at defined concentrations. The samples were then diluted 10,000-fold prior to the assays.

### Dipstick SELFIA of CRP

SELFIA of CRP was performed using a sandwich immunoassay format, as previously described.^11^ Briefly, a 4 mm-wide NC membrane was mounted onto a low-fluorescence backing card, with an absorbent pad affixed at one end of the membrane to form a ∼8 cm-long strip. To create the test line, 1.5 μL of pAb-P17 was dispensed at the center of the NC membrane and allowed to air-dry. The membrane strip was then prewetted and blocked with 3% BSA in PBS to minimize nonspecific binding.

For antigen detection, 10 μL of pAb-P17-FND (10 ng/μL) was mixed with 100 μL of CRP solution in a 96-well microplate, and the mixture was incubated at 37 °C for 15 min to allow antigen–antibody conjugation. The prepared strip was then inserted into the well, with the NC membrane directly contacting the solution. Capillary action, driven by the absorbent pad at the far end, directed the sample flow along the membrane. In the presence of CRP, antigen–antibody–FND complexes were captured by the immobilized antibodies at the test line. The strip remained in the well for 1 h, then was removed and dried at 50 °C for 2 min prior to fluorescence measurement using a custom-built SELFIA reader.^8^

### ELISA of CRP

The assays were performed according to the manufacturer’s protocol. First, pentameric CRP was dissociated into monomers via acid treatment. Specifically, CRP was diluted to 40 ng/mL in a total volume of 100 μL, followed by the addition of 50 μL of 0.25 N HCl. After a 15 min incubation at room temperature, 50 μL of 0.25 N NaOH was added, and the mixture was incubated for an additional 3 min. Next, 50 μL of the pretreated sample with a concentration of 20 ng/mL was added to each well, followed by 50 μL of Antibody Cocktail. The plate was incubated for 1 h at room temperature with shaking. Wells were then washed three times with the provided wash buffer. Subsequently, 100 μL of TMB development solution was added to each well and incubated for 10 min in the dark. Finally, 100 μL of stop solution was added. The plate was placed in a microplate reader (Spark, Tecan), shaken for 1 min, and optical densities (ODs) were measured at 450 nm.

### Sandwich QEMFIA of IL-6

Monoclonal mouse IgG 1 anti-IL-6 antibodies (MAB206R) and biotinylated polyclonal goat IgG anti-IL-6 antibody (BAF206) were used for the sandwich assays of IL-6. The protocols were identical to those used for CRP, except for the following modifications: (1) the NC membrane was coated with NA; (2) the capture and detection antibodies were replaced with BAF206 and MAB206R, respectively (cf. Supporting Information for more details).

## Results and Discussion

As illustrated in Figure 1a, the sandwich QEMFIA procedure comprises four major steps: (1) incubate antigens with detection-antibody-conjugated FNDs in a 96-well microplate; (2) coat the NC membrane in a microfiltration manifold with capture antibodies, followed by blocking it with BSA; (3) capture antigen–antibody-FND conjugates as they flow through the membrane, then wash away unbound FNDs and excess reagents; (4) transfer the FND-captured NC membrane to a 24-well detection plate for MMF measurement. Notably, the assay is conducted simultaneously on two platforms: a standard 96-well microplate and a custom-designed microfiltration manifold fabricated from PMMA. The microfiltration manifold consists of a top sample plate and a bottom filtration plate, both featuring precision-lapped mating surfaces. Between these two plates is an unbacked lateral flow NC membrane and a laser-cut Parafilm gasket, which ensures a proper seal (Figure S1). The diameter of the holes at the centers of the wells and on the gasket is 1.5 mm. A peristaltic pump is connected to the microfiltration vacuum manifold to precisely and consistently draw reagents across the NC membrane through the wells. This controlled flow promotes the uniform accumulation of target molecules on the membrane and helps overcome the diffusion limitations typically associated with static binding assays such as ELISA.

Another key component of this assay is the use of rounded FNDs as the reporters. These nanoparticles, approximately 100 nm in diameter, were treated with molten salts to remove sharp edges and surface irregularities, yielding smoother, more uniform particles optimized for in vitro diagnostic applications.^11^ The NC membrane employed in this study features a pore size of 12 μm and a thickness of 105–140 μm.^27^ It markedly differs from the 0.22 μm or 0.45 μm NC membrane commonly used in conventional flow-through immunoassays.^20–25^ The larger pore size prevents nonspecific trapping of 100 nm FNDs in the NC membrane, thus reducing background signals. The sample solution flows through the membrane at a typical rate of 10 μL/min. This slow, controlled flow greatly enhances the interactions between liquid-phase and surface-bound reagents within a confined volume of less than 0.2 μL in the membrane. After the assay, the FND-captured NC membrane is transferred to a homemade 24-well acrylic plate using double-sided tape. The FNDs are finally detected on the plate in an inverted fluorescence microscope system using the MMF technique to reduce background interference (Figure 1b). Notably, this high-performance microscope is equipped with a motorized sample stage for microplate imaging, allowing for high-throughput detection of FNDs by MMF in automation mode.

The experiment started with a direct QEMFIA assay utilizing the biotin–avidin model. Specifically, 100 nm rounded FNDs were surface-coated with b-BSA by noncovalent conjugation. The b-BSA-FND conjugates were then captured by NA coated on the NC membrane as they passed through the holes of the microfiltration manifold (Figure 1a). An excess amount of NA (∼1 μg/mm^2^) was immobilized on the membrane to enable effective capture of b-BSA-FNDs (100 μL) during the flow. Figure 2a shows fluorescence images of the dots containing the captured FNDs. The images were acquired using a large-area LED as the excitation light source, with a central wavelength of 534 ± 30 nm and a beam diameter of ∼4 mm at the sample position. Signals were not distinguishable when the amounts of FNDs employed in the assay were less than 1 ng. Figure 2b displays the dispersed fluorescence spectrum of an NC membrane attached to a PMMA plate illuminated with the green LED light. The spectrum significantly overlapped with that of FNDs in the 600–750 nm wavelength range,^28^ which hindered the detection of FND-labeled antibodies at low antigen concentrations. Fortunately, this issue can be effectively resolved by leveraging the quantum properties of the NV^–^ centers in FNDs.

**Figure 2:**
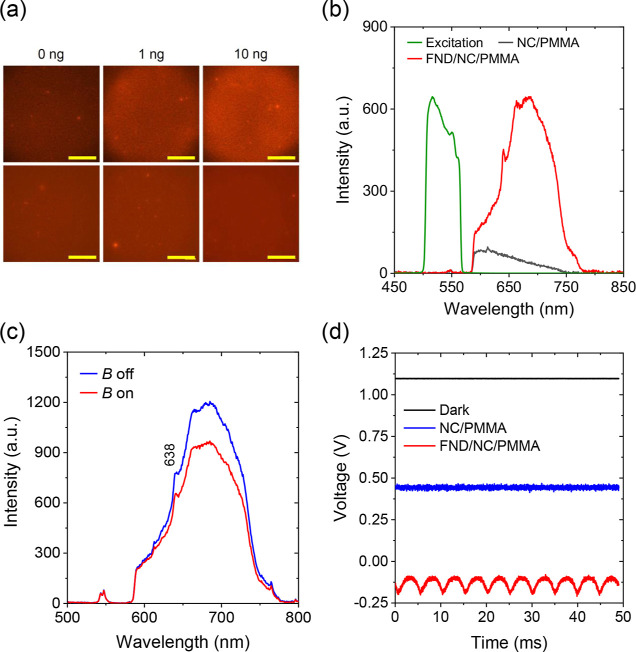
(a) Typical fluorescence images of b-BSA-coated FNDs captured on a NC membrane with (top) and without (bottom) NA immobilized on its surface. Scale bars: 500 μm. (b) LED excitation and dispersed fluorescence spectra of an NC membrane attached to a PMMA microplate (NC/PMMA) and b-BSA-FNDs captured on an NC membrane attached to a PMMA microplate (FND/NC/PMMA). The amount of FNDs used in the measurement was 100 ng. (c) Comparison of the fluorescence spectra of FND/NC/PMMA with and without an external magnetic field (*B* = 30 mT). (d) Signals measured for NC/PMMA and FND/NC/PMMA under LED excitation and a modulated magnetic field using a photomultiplier tube. The voltage differences between blue and black traces represent NC/PMMA signals, while the red-blue differences correspond to FND signals. The FND fluorescence intensities were modulated at 204.8 Hz.

To incorporate the MMF function into the microscope system for real-world immunoassay applications, we installed a small electromagnet (35 mm diameter) on the diascopic illuminator positioned above the sample stage of the fluorescence microscope (Figure 1c). A homemade current amplifier powered the electromagnet. The typical separation between the electromagnet’s top surface and the sample plate’s bottom was 1 mm (Figure 1b). The magnetic field strength at the sample position was about 30 mT, which significantly reduced the total fluorescence intensities of the FNDs captured on the NC membrane by more than 10% (Figure 2c). By applying an alternating electric current at 102.4 Hz to the electromagnet, the magnetic field was switched, resulting in modulation of the fluorescence signals from the FNDs at 204.8 Hz (Figure 2d). The intensity modulation depth was 13%, in agreement with our previous measurements.^8^ In contrast, nearly no intensity changes (<0.01%) were observed for the NC membrane and the PMMA plate under the same conditions. With this MMF method, we have been able to eliminate background fluorescence signals from the NC membrane and the PMMA plate, clearly revealing the characteristic spectra of the NV^–^ defects using a fast Fourier transform method (cf. Figures S2 and S3 and Supporting Information for further analysis).^8^ The capability of performing the modulation at a frequency exceeding 100 Hz indicates the high responsivity of this MMF detection system.

Figure 3a,b present, respectively, the sensitivity enhancement of the MMF method and the dynamic range of the direct QEMFIA assay using the biotin–avidin model. The control experiment of this assay employed an NC membrane coated with BSA instead of NA. As shown in both figures, signal intensities in the test experiments increased linearly with b-BSA-FND concentration. In contrast, in the control experiments, the signals were negligible even with a 1000-fold increase in b-BSA-FND concentration. Nonspecific binding accounted for ∼0.5% of the total signal. A distinct advantage of the microfiltration method is that all the b-BSA-FNDs were confined within a detection area of ∼2 mm^2^ for MMF detection. This allows us to detect ∼10 pg of 100 nm FNDs, corresponding to ∼5 × 10^3^ spherical particles, on the NC membrane. The sensitivity of this immunodiagnostic platform is expected to be further improved by reducing the detection area and refining the shapes and surface properties of these diamond nanocrystals.^11,29–31^

**Figure 3:**
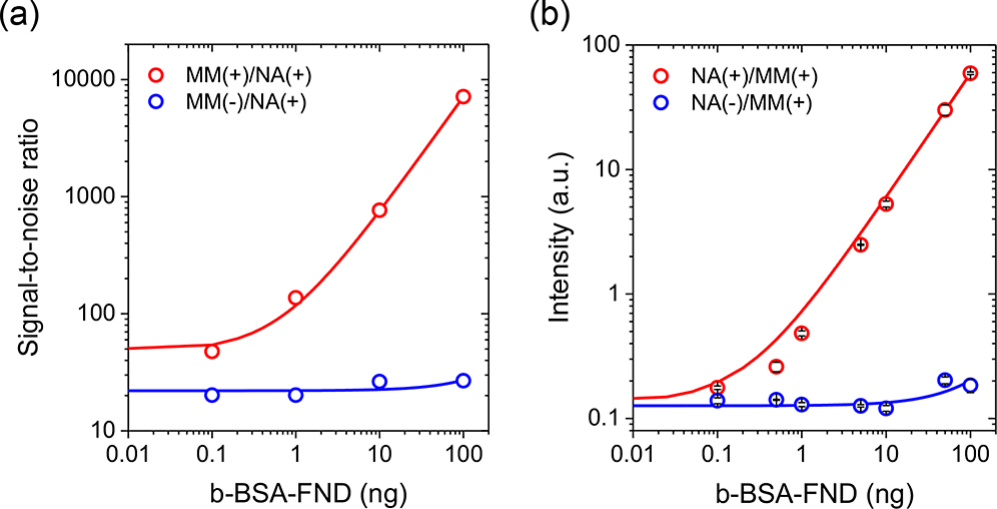
(a) Signal-to-noise ratios of direct QEMFIA for the biotin–avidin model with and without magnetic modulation, denoted as MM­(+) and MM(−). NA: NeutrAvidin on NC membranes. (b) Dose dependence of the fluorescence intensities of b-BSA-FNDs captured on NC membranes coated with and without NA, denoted as NA­(+) and NA(−). The solid lines represent the best linear fits of the experimental data plotted on linear scales.

Next, we evaluated QEMFIA’s performance for CRP, a protein produced by the human liver in response to inflammation.^32^ The protein consists of 5 identical subunits (molecular weight of 23 kDa/each)^33^ and can be targeted by multiple antibodies simultaneously. Its concentration in the bloodstream typically ranges from <5 μg/mL in healthy individuals to up to 500 μg/mL during acute inflammation. The wide variation in concentration and its clinical significance make this protein an ideal test system for this study. Based on our previous investigations with spin-enhanced LFIA (or SELFIA in short),^11^ we prepared the CRP solution in PBS and performed sandwich QEMFIA using 100 nm FNDs as the reporters. The MMF technique described above was then applied to detect pAb-P17-conjugated FNDs carrying CRP, which were captured by pAb-P17 antibodies immobilized on the NC membrane.

Figure 4a shows the results of the sandwich QEMFIA for CRP by using a 100 μL sample and 200 ng FNDs. The assay demonstrated a working concentration range of 0.01–10 ng/mL. The limit of detection (LOD), defined as 3× the standard deviation (SD) of the blank signal, was ∼9 pg/mL (or ∼70 fM or ∼4 × 10^7^ molecules/mL), where each FND may carry only one CRP molecule. Notably, both the sensitivity and dynamic range surpassed those of SELFIA by 10-fold under the same experimental conditions (Figure 4b). In comparison, ELISA achieved a LOD of ∼50 pg/mL by using only 50 μL of CRP as the sample (Figure 4c); however, it required acid treatment to dissociate pentameric CRP into monomers prior to analysis. From this perspective, QEMFIA clearly offers an advantage in time efficiency. Additionally, QEMFIA also provides a flexible detection range that can be readily adjusted by varying the sample volume and FND quantity. For example, the working concentration range can be shifted to 1–100 ng/mL with just a 10 μL CRP sample and 100 ng FNDs (Figure 4a). This flexibility enables easy adaptation to various experimental conditions, simplifying its use in practical settings.

**Figure 4:**
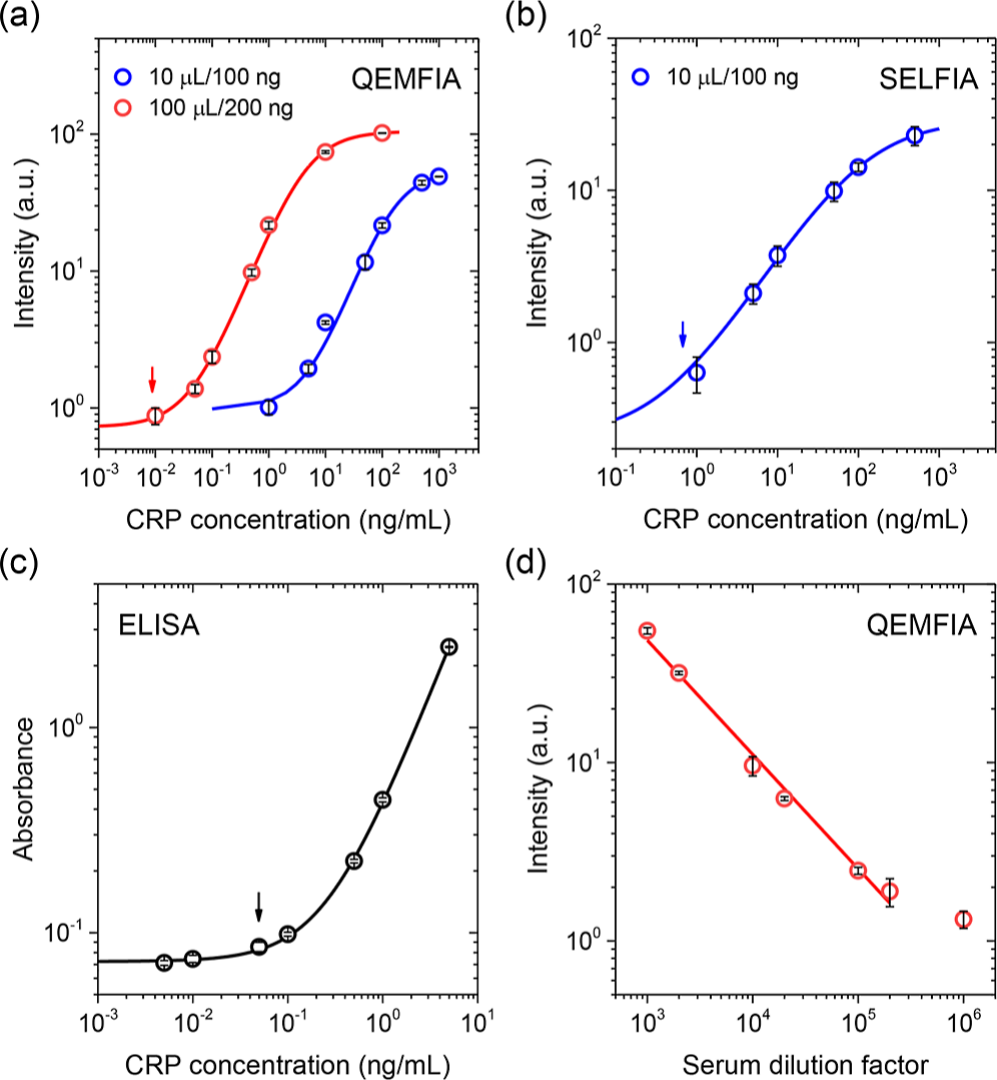
(a) Sandwich QEMFIA of CRP. The detection range of QEMFIA is shifted by controlling the sample volume (μL) and the amount of FND (ng) used. (b) Sandwich SELFIA of CRP, performed with 10 μL of sample and 100 ng FND. (c) Sandwich ELISA of CRP. The solid lines in (a–c) are best fits of experimental data to a logistic model, *y* = *a* + (*b* – *a*)/(1 + (*x*/*c*)^*d*^), where *a*, *b*, *c*, and *d* are constants. (d) Linearity-of-dilution assessment of sandwich QEMFIA for CRP using a human serum sample. All data are presented as mean ± SD (*n* = 3), and arrows indicate the LODs of the individual assays: (a) 9, (b) 101, and (c) 50 pg/mL.

To assess the clinical applicability of sandwich QEMFIA, a linearity-of-dilution experiment was conducted using human serum.^34,35^ We first diluted serum samples from 1:1000 to 1:1,000,000 in 3% BSA/PBS, after which the assay’s dilution linearity was measured with 100 μL of sample and 200 ng of FNDs as reporters. A strong linear correlation between signal intensity and dilution factor was observed, with an *R* ^2^ value of 0.990 over the dilution range of 1:1000 to 1:200,000 (Figure 4d). However, the ultimate detection sensitivity is limited to approximately 10 pg/mL due to abundant serum proteins (such as human serum albumin and transferrin)^36^ that interfere with measurements and increase nonspecific binding to ∼2% (cf. Supporting Information for more discussion).

To further evaluate the assay’s validity, a spike-and-recovery experiment was performed. We first determined the concentration of CRP in the serum to be 7.5 μg/mL by diluting the sample 10,000-fold prior to measurement. Recovery rates of 105% and 111% were obtained for the diluted serum spiked with 0.5 and 2.0 μg/mL CRP, respectively (Table 1). Collectively, these results demonstrate the high accuracy and precision of QEMFIA in analyzing complex biological matrices, including human serum.

**Table 1:** Recovery of CRP Spiked in Human Serum by QEMFIA^a^

spiked level (μg/mL)	calculated concentration (μg/mL)	measured concentration (μg/mL)	recovery (%)
0.5	8.0	8.4	105
2.0	9.5	10.5	111

a The initial CRP concentration in the serum was 7.5 μg/mL.

IL-6 is a multifunctional cytokine that plays a pivotal role in regulating immune responses, inflammation, and hematopoiesis.^37^ In healthy individuals, circulating IL-6 levels are generally low, typically below 5 pg/mL.^38^ However, in the presence of pathological conditions such as infection, trauma, autoimmune disorders, or cancer, IL-6 levels can rise dramatically, making it a valuable biomarker for early detection and disease monitoring. Due to its low baseline concentration and rapid elevation during inflammatory responses, enhancing QEMFIA’s sensitivity is crucial for detecting even minimal changes in IL-6 levels. This is achievable by leveraging the biotin–avidin interactions using biotinylated antibodies and NA-coated NC membrane to increase the antigen capture efficiency,^7^ as illustrated in Figure 5a and detailed in the Experimental Section. Employing this optimized strategy enabled us to achieve an LOD of ∼2 pg/mL (or ∼80 fM) for IL-6 (Figure 5b). Following the protocols of CRP, a linearity-of-dilution experiment was also performed for this disease marker in human serum. The result is presented in Figure S4, showing the reliability of this QEMFIA platform.

**Figure 5:**
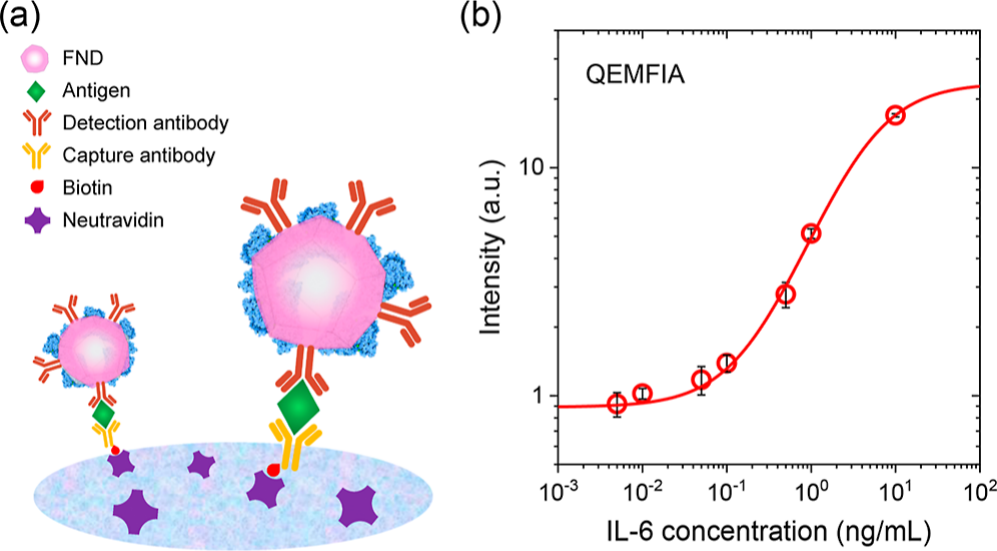
(a) Illustration of FNDs captured on the NC membrane via biotin–avidin interactions. Different from the workflow shown in Figure 1a, biotinylated capture antibodies and NA-coated NC membrane are used in the assays. (b) Sandwich QEMFIA of IL-6 using 600 μL sample and 100 ng FNDs. Data are presented as mean ± SD (*n* = 3).

Table S1 presents a comparative analysis of the performance of QEMFIA, ELISA, dot blot, and SELFIA for CRP. The first three methods all offer high-throughput capabilities. However, the dot blot assay is a semiquantitative technique that provides only an approximate measure of target concentration (Figure S5 for the results and Supporting Information for experimental details). Compared to ELISA, QEMFIA offers a broader dynamic range, making it better suited for applications that require a wide dynamic range while maintaining high sensitivity. Moreover, QEMFIA eliminates the need for extensive washing steps and directly measures FND fluorescence on the membrane via magnetic modulation and lock-in detection. This significantly reduces assay time compared to ELISA (<1 h versus >3 h). In essence, QEMFIA integrates the strengths of both dot blot and ELISA by using FNDs as quantum reporters. The integration streamlines the overall immunoassay workflow, enhancing both efficiency and practicality.

Finally, it is worth noting that although the 100 nm bioconjugated FND particles could not be detected individually on the NC membrane because of the high background, they are readily visible on clear polystyrene beads when examined under a confocal fluorescence microscope. Figure 6a–c present two- and three-dimensional bright-field and confocal fluorescence images of these particles, demonstrating the feasibility of their visualization and spatial characterization (cf. Supporting Information for experimental details). Further enhancement in detection sensitivity is anticipated through the use of larger FNDs or similarly sized particles with a greater number of NV centers implanted into the diamond matrix.^7^

**Figure 6:**
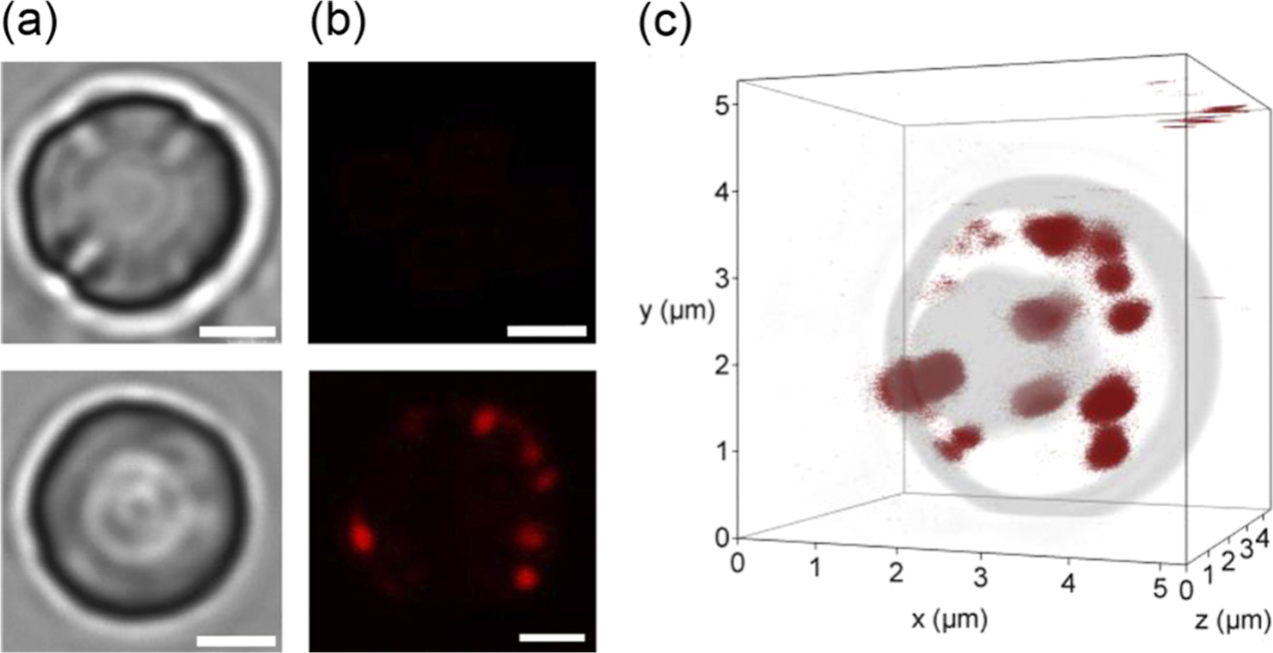
(a) Bright-field and (b) confocal fluorescence images of a bare streptavidin-coated polystyrene bead (top) and a streptavidin-coated polystyrene bead conjugated with b-BSA-coated FNDs (bottom). Scale bars: 1 μm. (c) Three-dimensional confocal fluorescence image of b-BSA-coated FNDs attached to a streptavidin-coated polystyrene bead. Each red dot represents a single FND particle of ∼100 nm in diameter.

## Conclusion

We have conducted quantum spin detection and introduced an innovative immunoassay platform that harnesses the distinctive quantum characteristics of NV^–^ centers in FNDs. The QEMFIA platform integrates quantum sensing technologies with microfiltration-based methods to deliver exceptional speed, sensitivity, and throughput, surpassing the performance of conventional assay systems. It demonstrates much promise for the detection of disease biomarkers and can be utilized across a broad range of biomedical applications, including the identification of pathogenic bacteria and tumor cells. Given its ability to provide rapid and accurate diagnostic results with high sensitivity, QEMFIA stands to significantly enhance clinical health monitoring. This work marks a pioneering step toward translating quantum technology into real-world healthcare solutions.

## Supplementary Material





## Notes

Chemicals and materials, QEMFIA apparatus, MMF spectra, serum dilution assay, dot blot immunoassay, single FND detection, nonspecific binding, and performance comparison between four different immunoassays (PDF)
Automated fluorescence intensity measurements for FNDs in QEMFIA (MP4)
