# Supplementary material for: Quantum Spin Detection in Microfiltration Immunoassays for Ultrasensitive and High-Throughput Diagnostics
Source: Anal Chem. 2026 Feb 6;98(6):4562–70. doi: 10.1021/acs.analchem.5c05569 (PMC12921671; doi:10.1021/acs.analchem.5c05569)
Supplement: Supplementary file 1 [file ac5c05569_si_001.pdf]

Supporting Information

Quantum Spin Detection in Microfiltration Immunoassays for Ultrasensitive and High-Throughput Diagnostics

Trong-Nghia Le,[†] Xuan Mai Lam,[†] Yi-Xiu Tang,[†] Yuen Yung Hui,[†] An-Jie Liu,[†] and Huan-Cheng Chang[†,‡,§,*]

[†]*Institute of Atomic and Molecular Sciences, Academia Sinica, Taipei City 106, Taiwan*

[‡]*Department of Chemical Engineering, National Taiwan University of Science and Technology, Taipei City 106, Taiwan*

[§]*Department of Chemistry, National Taiwan Normal University, Taipei City 106, Taiwan*

Table of Contents

1. Experimental Section

1.1 Chemicals and materials

1.2 Magnetically modulated fluorescence spectroscopy

1.3 Dot-blot immunoassay of CRP

1.4 Sandwich QEMFIA of IL-6

1.5 Single FND detection

2. Results and Discussion

2.1 Fluorescence spectra of FNDs

2.2 Nonspecific binding

3. References

4. Tables

5. Figures

1. Experimental Section

1.1 Chemicals and materials

Bovine serum albumin (BSA) and phosphate-buffered saline (PBS) were sourced from Sigma-Aldrich and Gibco, respectively. NeutrAvidin biotin-binding protein and biotinylated BSA (b-BSA) were obtained from Thermo Fisher Scientific. Polyclonal anti-CRP rabbit antibodies (pAb-P17, GTX72817) were purchased from GeneTex, and native CRP antigens were supplied by EastCoast Bio. Human C Reactive Protein ELISA Kits (ab99995) were obtained from Abcam, and human serum from human male AB plasma, USA origin (H4522), was purchased from Sigma Aldrich. Human IL-6 protein was purchased from PeproTech. Monoclonal mouse IgG 1 anti-IL-6 antibodies (MAB206R) and biotinylated polyclonal goat IgG anti-IL-6 antibody (BAF206) were obtained from R&D Systems. Syringe filters (25 mm diameter each), made of polyester/PET material and featuring a 1.2 μm porosity, were sourced from Macherey-Nagel. Streptavidin-coated polystyrene beads (3 μm in diameter) were purchased from Bangs Laboratories. All other chemicals were used as received from Sigma-Aldrich.

1.2 Magnetically modulated fluorescence spectroscopy

Dispersed fluorescence spectra of FNDs deposited on the NC membrane were acquired using a photonic multichannel analyzer (C7473, Hamamatsu) attached to the eyepieces of the inverted fluorescence microscope. Fluorescence signals were captured with and without magnetic modulation and detected by the multichannel analyzer. The exposure time of each measurement was 100 ms, and the magnetic field was modulated at a frequency of 2 Hz. Time-varying signals were obtained at each wavelength as the magnetic field switched directions over 100 s. An OriginLab program analyzed the time evolution of the signals using Fast

Fourier Transform (FFT) to obtain demodulated fluorescence intensities. The FND spectra were finally restored by plotting the intensities against their corresponding wavelengths.

1.3 Dot blot immunoassay of CRP

Purified CRP protein was first diluted in PBS to a working stock concentration of 1.67 mg/mL. From this stock, a series of five-fold serial dilutions was prepared in PBS to yield final concentrations of 1.67, 0.33, 0.067, 0.0134, and 0.0027 μg/μL. For dot blotting, 30 μL of each diluted solution was applied to a Bio-Dot apparatus (Bio-Rad) under vacuum, corresponding to protein loads of 50, 10, 2, 0.4, and 0.08 ng per spot, respectively. The membrane was then dried in a 50 °C incubator and blocked with 5% non-fat milk in PBS containing 0.05% Tween-20 (PBS-T) for 1 h at room temperature. After blocking, the membrane was incubated with a 1:100 dilution of rabbit anti-CRP antibody (GeneTex) in PBS for 30 min at room temperature. Following three washes in PBS-T, the membrane was further incubated with a 1:1500 dilution of horseradish peroxidase (HRP)-conjugated donkey anti-rabbit IgG secondary antibody (Cytiva) for 30 min. The signals were developed using ECL Western blotting detection reagents (Cytiva) and visualized with an imaging system (iBright, Thermo Fisher Scientific).

1.4 Sandwich QEMFIA of IL-6

Protocols include: (1) Add BAF206 antibody (0.01 mg/mL, 2 μL) to IL-6 solution (600 μL) with varying concentrations in the individual wells of a 96-well microplate, and incubate for 15 min. (2) Assemble the microfiltration manifold and connect it to a peristaltic pump. (3) Add NA (1.5 μL, 1 mg/mL) into the individual wells of the microfiltration manifold and allow it to air dry for 5 min. (4) Add 3% BSA/PBS (100 μL) in the individual wells, and switch on the peristaltic pump to facilitate the flow of the solution at a rate of about 20 μL/min through the membrane to fully block unbound sites on the NC membrane. (5) Transfer the BAF206-

IL-6 solution prepared in Step 1 to the microfiltration manifold, and switch on the peristaltic pump to pull the reagents across the NC membrane at a rate of about 10 μL/min. (6) Add MAB206R-FND suspension (100 μL, 1 μg/mL) to the microfiltration manifold, maintaining a flow rate of 10 μL/min. (7) Wash the membrane with 1% BSA/PBS (200 μL) at a flow rate of about 20 μL/min. (8) Transfer the NC membrane with FNDs captured to the homemade plate using double-sided tape. (9) Measure MMF of FNDs on an inverted fluorescence microscope equipped with an electromagnet.

1.5 Single FND detection

Streptavidin-coated polystyrene beads (3 μm in diameter) were first washed twice with PBS according to the manufacturer's instructions. The beads (5×10^6 particles, 100 μL) were then mixed with 100 μL b-BSA-conjugated FNDs (2 ng μL) and incubated for 2 h. Subsequently, the mixture was centrifuged at 5000 *g* for 5 min and the pellet was washed twice with PBS to remove unbound FND particles. The pellet was then resuspended in PBS and drop-casted on a glass slide. Time-gated confocal fluorescence imaging was conducted using an inverted microscope system (SP8, Leica) with the excitation light set at 532 nm. Fluorescence emission was collected through an oil-immersion objective (63×, NA 1.4) and detected by a photomultiplier tube at 650 – 800 nm. The gating time the was set at 10 ns.

2. Results and Discussion

2.1 Fluorescence spectra of FNDs

To further illustrate the power of the MMF method in removing background signals, we obtained the dispersed fluorescence spectra of FNDs on the NC membrane with and without the magnetic fields. In **Figure 2b**, we present the fluorescence spectra of the substrates excited by the 534±30 nm LED light source equipped in the inverted fluorescence microscope. With

100 ng of FNDs deposited on the NC membrane, the fluorescence spectrum showed an intensity maximum at 685 nm, corresponding to the phonon sidebands of the electronic transition ($^3A_2 \rightarrow {}^3E$) of NV^- centers.[S1] However, the NC membrane also emitted fluorescence over the 550 – 750 nm wavelength range. The strong overlap between these two spectra hindered the detection of FNDs at quantities below 1 ng. Magnetic modulation provides a solution to this problem.

Figure S2a displays a typical time trace of the fluorescence signals of FND/NC/PMMA at 685 nm with magnetic modulation. The FND particles showed a 20% modulation in fluorescence intensity when subjected to a 30 mT magnetic field switching directions at 2 Hz. Fast Fourier transform (FFT) of the time-varying signals revealed a fundamental frequency peak centered at 4 Hz (**Figure S2b**), with its height corresponding to the demodulated fluorescence intensity. The frequency was doubled due to the spin-triplet ground state ($^3A_2$) of the NV^- centers and the random orientation of the FND particles on the membrane. With this method, an intrinsic fluorescence spectrum of the NV^- centers could be obtained by plotting the demodulated fluorescence intensities against wavelength, as shown in **Figure S3a**. As noted, this spectrum was free of the signals from the background interference due to photoexcitation of the NC membrane and the surrounding PMMA well (**Figure 2b**). **Figure S3b** compares the spectrum with the fluorescence spectrum of FNDs dispersed on a glass coverslip and obtained using laser excitation at 594 nm, which generates fluorescence signals from NV^- centers only. The agreement is satisfactory, aside from discrepancies arising from differences in the filters used in the light paths of the two measurements.

We note that the results shown in **Figure 2d** were obtained by collecting total fluorescence from FNDs deposited on an NC membrane using a photomultiplier tube. These measurements could be readily performed at frequencies exceeding 100 Hz. However, to acquire the fluorescence spectra of FNDs, a spectrometer equipped with a CCD camera is

required. In this case, the modulation frequency must be reduced to, for example, 2 Hz to match the CCD frame rate. Subsequently, an FFT is applied to the time-varying signal at each wavelength to reconstruct the background-free spectra.

2.2 Nonspecific binding

A major limitation to the detection sensitivity of QEMFIA is the nonspecific binding between proteins. In the direct assay using the biotin-avidin model as shown in **Figure 2b**, the nonspecific binding between b-BSA-conjugated FNDs and the NA-coated NC membrane contributed approximately 0.5% to the total signal. For sandwich QEMFIA, the value may vary, depending on the antigen-antibody pairs used in the assay. The typical value was 1% if the assay was performed in 3% BSA/PBS. However, the contribution of nonspecific binding could increase to more than 2%, as measured for the human serum sample diluted 1,000,000-fold using 3% BSA/PBS (**Figure 4d**). Under this dilution condition, the CRP concentration was reduced to 7.5 pg/mL, below our detection limit. Therefore, the observed signals were mainly contributed by nonspecific binding. In human serum, the normal total protein level is 60–80 g/L.[S3] Following a 1,000,000× dilution with PBS buffer, the level decreases to 60–80 ng/mL, which remains 3 orders of magnitude higher than the corresponding CRP concentration. The presence of abundant serum proteins, such as human serum albumin and transferrin, may interfere with the evaluation of assay specificity for this quantum-enabled platform.

3. References

S1. Manson, N. B.; Hedges, M.; Barson, M. S. J.; Ahlefeldt, R.; Doherty, M. W.; Abe, H.; Ohshima, T.; Sellars, M. J. NV−-N+ pair centre in 1b diamond. *New J. Phys.* **2018**, *20*, 113037.

S2. Yang, T.-I.; Huang, Y.-W.; Bista, P.; Ding, C.-F.; Chen, J.; Chiang, C.-T.; Chang, H.-C. Photoluminescence of nitrogen-vacancy centers by ultraviolet one- and two-photon excitation of fluorescent nanodiamonds. *J. Phys. Chem. Lett.* **2022**, *13*, 11280-11287.

S3. Busher, J. T. Serum Albumin and Globulin. In *Clinical Methods: The History, Physical and Laboratory Examination*, 3rd edition; Walker, H. K., Hall, W. D., Hurst, J. W., Eds.; Boston: Butterworths; 1990; Chapter 101.

4. Tables

Table S1. Performance comparison between four different immunoassays for CRP.

Parameters	QEMFIA	ELISA	Dot Blot	SELFIA
Analyte	Pentameric CRP	Monomeric CRP	Pentameric CRP	Pentameric CRP
Molecular weight	115 kDa	23 kDa	115 kDa	115 kDa
Reporter	FND	Enzyme	Enzyme	FND
Sample volume	100 µL	50 µL	30 µL	100 µL
Wash	1 time	3 times	3 times	Not required
Total assay time	~1 hour	~3 hours	~3 hours	~1 hour
Assay format	24-well microplate	96-well microplate	96-well microplate	Single strips
Limit of detection	0.01 ng/mL	0.05 ng/mL	Semi-quantitative	0.1 ng/mL
Dynamic range	~3 orders	~2 orders	Semi-quantitative	~2 orders
Achievable throughput	~24 samples/h[a]	~32 samples/h[b]	~32 samples/h[b]	~24 samples/h[c]
Automation	+	+	–	–

[a]Experiments run in all 24 wells.

[b]Experiments run in all 96 wells.

[c]Experiments run with 24 strips in parallel.

5. Figures

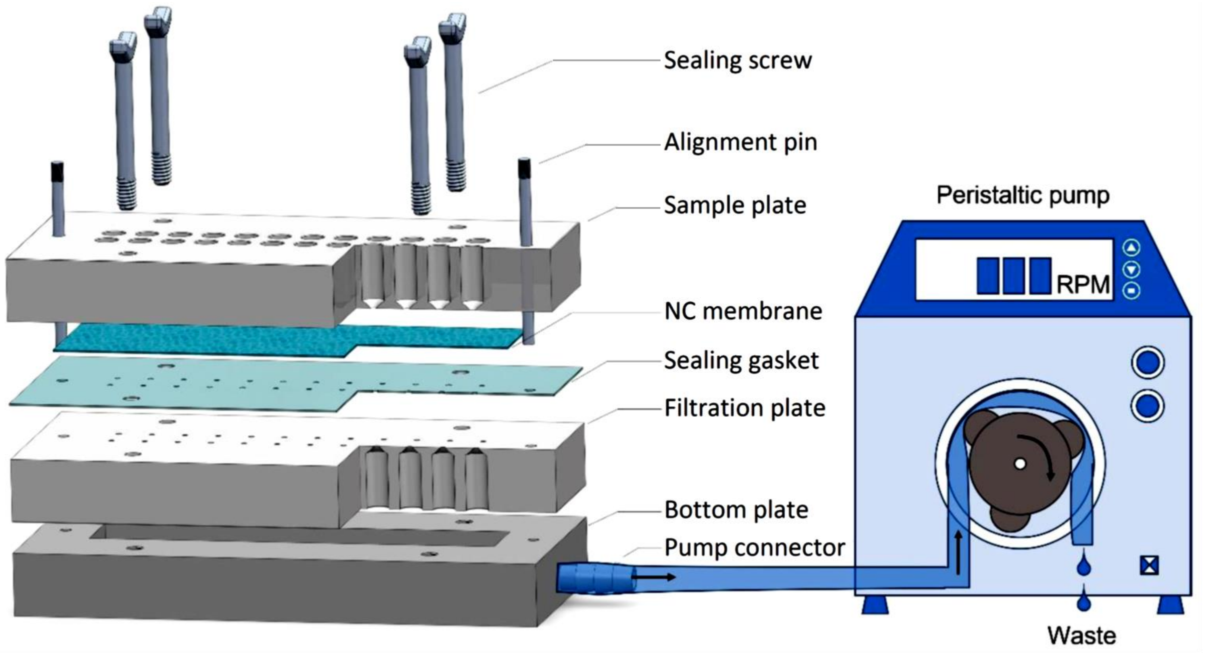

Figure S1. Structure of the QEMFIA apparatus. To facilitate liquid flow and reduce variations in flow rate, absorbent pads commonly used in lateral flow immunoassays are additionally placed in each well of the filtration plate, which is connected to a bottom plate and a peristaltic pump.

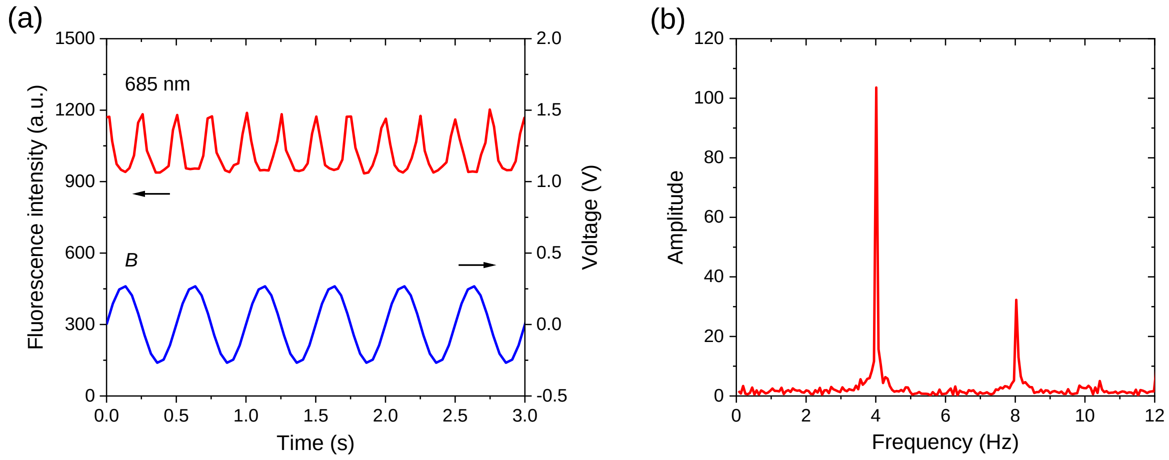

Figure S2. (a) Time trace of the peak fluorescence intensity at 685 nm under 534-nm LED excitation and magnetic modulation at 2 Hz. The blue curve shows the applied magnetic field (*B*) measured with a Gaussmeter. (b) Frequency spectrum of the modulated fluorescence intensities in (a) after FFT.

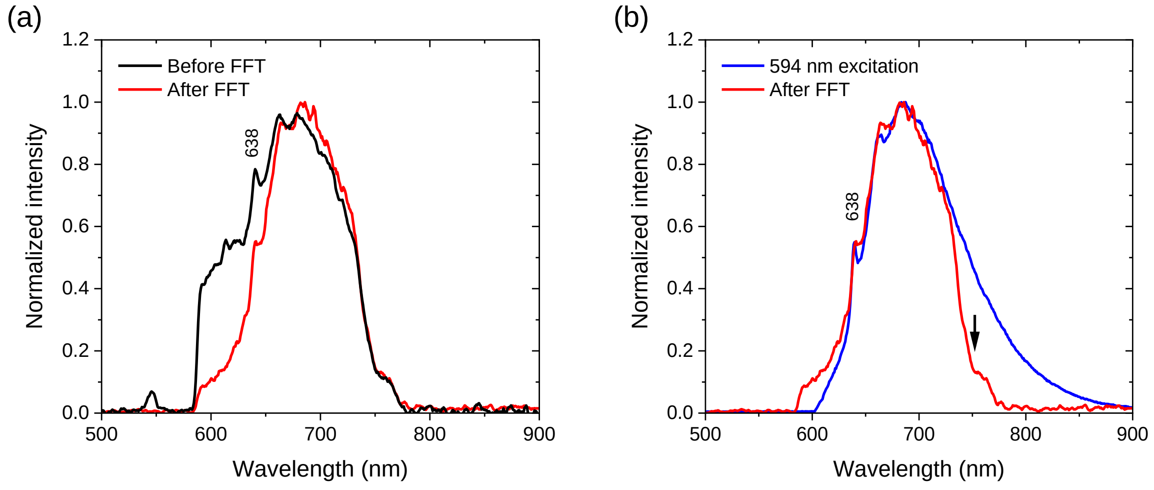

Figure S3. (a) Fluorescence spectra of FND/NC/PMMA before and after FFT analysis as described in the text. (b) Normalized fluorescence spectra of NV^- centers in FNDs deposited on a glass cover slip and an NC membrane. The former was obtained by laser excitation at 594 nm, whereas the latter was acquired by LED excitation (534±30 nm) and magnetic modulation (2 Hz), followed by FFT analysis. The black arrow indicates the cutoff wavelength of the filters used in the fluorescence measurement on an inverted microscope.

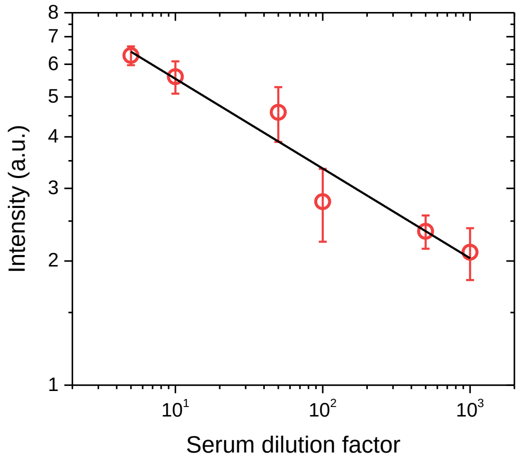

Figure S4. Linearity-of-dilution assessment of sandwich QEMFIA for IL-6 using a human serum sample. Data are presented as mean ± SD (n = 3). The initial IL-6 concentration was 50 ng/mL.

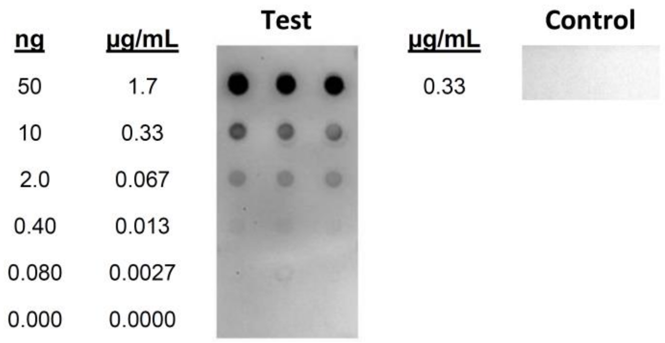

Figure S5. Dot-blot immunoassay of CRP. Each assay used 30 µL of the sample solution, and the LOD is ~10 ng/mL. The control experiment consisted of 0.33 µg/mL CRP but without secondary antibodies.
